# Supervised mHeath Exercise Improves Health Factors More Than Self-Directed mHealth Exercise: A Clinical Controlled Study

**DOI:** 10.3389/fpubh.2022.895474

**Published:** 2022-08-05

**Authors:** Yukun Hu, Yong Zhang, XiaoYa Qi, XiaoYang Xu, Jamal Rahmani, Ruixue Bai, Ying Mei

**Affiliations:** ^1^Department of Health Management, The Second Hospital Affiliated to Chongqing Medical University, Chongqing, China; ^2^School of Public Health and Health Management, Chongqing Medical University, Chongqing, China; ^3^Cancer Research Center, Shahid Beheshti University of Medical Sciences, Tehran, Iran

**Keywords:** mHealth, exercise, obesity, overweight, heartbeat tracker

## Abstract

Wearable physical activity trackers are getting popular for the self-management of weight despite limited evidence of their efficacy. Studies have proven that on-site supervised exercise is superior to unsupervised exercise for many health problems, there is no evidence comparing the effectiveness of remote supervision exercise with self-directed exercise based on mHealth. This study aims to compare the effects of mHealth-based supervised exercise to mHealth-based self-directed exercise on weight control in the overweight and obese population. A nonrandomized controlled clinical study was conducted. Overweight or obese volunteers were given personalized exercise prescriptions based on their HRR (Heart Rate Reserve), all patients were equipped with wearable heartbeat trackers to follow their exercise performance and additional remote supervisions were added to the intervention group. Exercise performances, weight losses, and health examinations were compared between 2 groups after 12 weeks of follow-up. Analysis of covariance (ANCOVA) was used to determine any differences between study groups after intervention. Two groups had the same rate of attrition in 12 weeks of follow-up, but the exercising day, the effective exercising day and the rate of effective exercising day in the supervised group were higher than those in the control group. Weight loss was −2.7 ± 2.8 kg in the intervention group and −2.0 ± 2.9 kg in the control group (*P* = 0.23). Compared with the control group, participants in the intervention group improved their liver function, kidney function, fasting blood sugar, total cholesterol, and triglyceride. mHealth-based supervised exercise is more effective in health factors improvement than mHealth-based self-directed exercise among overweight and obesity participants.

## Introduction

Overweight and obesity, as one of the leading risks for death and disability globally in 2019 (1), have become a major public health problem in China and around the world. The recent China national data showed that nearly 50% of adults were overweight or obese and the trend is still going upward (2).

Many studies have shown that taking enough physical activity is an effective way for weight loss and other health benefits (3–5). In addition, literature has also shown that supervised exercise by offering encouragement and motivation resulted in a higher adherence and more health benefits than unsupervised exercise for people with obesity (6), diabetes (7), and intermittent claudication (8). However, most of the studies about supervised exercise were on-site in clinic settings with face-to-face instructions, which makes it costly, resource-intensive, and difficult to implement at other venues. Therefore, in community settings, the prescription of exercise therapy is usually as simple as “go home and walk.”

Mobile health (mHealth) is defined by the WHO as medical or public health practices supported by mobile devices, such as mobile phones, wearable devices, and other personal wireless devices (9). Physical activity can be monitored by using wearable trackers, such as pedometers, accelerometers, and heart-rate trackers (10). For the efficacy of wearable devices on obesity, two systematic reviews suggested may be a better option than a standard weight loss program (11, 12). However, as commercialized physical activity trackers are getting more and more popular for self-management, one scholar who said “wearable devices as facilitators, not drivers of health behaviors”(13), believed that wearable devices alone have limited effects on behaviors engagement like exercise. This may be true because Jakicic et al. found wearable trackers may not offer an advantage over standard behavioral weight loss approaches (14). Therefore, the combination of supervised exercise with self-directed exercise may be a promising strategy for weight management.

In fact, data generated by wearable devices can be used not only for customers to self-manageme but also for professionals to supervise exercise remotely. However, many studies on wearable devices with professional supervision and feedback had minimal or standard care as control (15), which made it impossible to estimate the effects of wearable devices-based remote supervision while wearable devices-based self-directed exercise was involved in the intervention group at the same time.

As supervision can be realized by expanding the using of data from wearable devices, introducing remote supervision into the mHealth-based weight control program might be a useful strategy to enhance the effects of weight control. However, the specific effects of remotely supervised exercise on weight control have not been fully documented. This study is therefore aimed to investigate the clinical effects of heartbeat tracker-mediated remotely supervised exercise on overweight and obesity.

## Methods

### Study Design

This study is a prospective, nonrandomized controlled study approved by the Ethics Committee of Chongqing Medical University (s2018-065-01). Eligible participants were recruited and informed for consent to take part. Due to limited wearable devices, intervention and control trials were conducted one by one.

### Participants

Overweight/obese residents in Chongqing city were recruited. A recruitment advertisement was posted on the official website of the Second Affiliated Hospital of Chongqing Medical University. When a candidate came to the hospital with an appointment, an interview was conducted to evaluate the eligibility for inclusion. The inclusive and exclusive criteria were as follows:

Inclusive criteria: (1) man or woman (2); age between 18 and 65; (3) 24 ≤ body mass index (BMI) <40; (3) no habit of routine exercise; (4) no plan to join other exercise programs during the trial; (5) not on a diet for weight loss; (6) not taking a drug for weight loss; and (7) low to medium risk of cardiocerebrovascular disease.

Exclusive criteria: (1) wearing a heart pacemaker, peripheral nerve stimulator, insulin pump, intravascular stent, or metal heart valve; (2) mental or body disability to follow instructions; (3) pregnancy or plan to be pregnant during the trial; and (4) severe arrhythmia, Brugada syndrome, and other systemic diseases.

Recruitment was divided into two stages. Candidates recruited from 2019-7 to 2019-10 were rolled in the intervention group, while others recruited from 2019-11 to 2019-12 were rolled in the control group.

Two regular health examinations (at the baseline and the endpoint) were provided to all eligible participants as incentives (worth 50 dollars).

### Intervention

The intervention had three core components of management: (1) a personalized exercise prescription; (2) a heartbeat tracker and paired mobile phone App; and (3) an online chatroom.

The personalized exercise prescription was based on each person's Heart Rate Reserve (HRR), effective exercising was defined as the heartbeat rate reaching 40–60% of HRR [i.e., moderate-intensity exercise (16) for at least 30 min cumulatively per exercise session]. Participants were required to do effective exercise at least 3 days per week, without limitation of exercise types.

A bracelet (Mio-Fuse) that can trace heartbeat was lent to participants. the paired App which can retrieve data from the bracelet by Blue-tooth™ was installed on the participant's smartphone. Participants were instructed to wear and turn on the bracelet when exercising. Visualized feedback of exercise performances and historic records are provided by App to participants. When this App was running, exercise data were automatically synchronized to the cloud server by the network for researchers to access by a computer station and make personalized feedback accordingly.

An exclusive online chatroom that supports multimedia messages, such as text, pictures, audio, and short video, was created in the Wechat App (a smartphone application of Tencent Company, China) for each group. Researchers in the intervention group chatroom were active to instruct, remind and encourage participants to follow exercise prescriptions based on their daily exercise data. Participants were also encouraged to share their exercise accomplishments and experiences in their chatroom. The mHealth system used in this study is illustrated in Table 1.

**Table 1:** The structure and components of supervised mHealth exercise.

**Terminal**	**Devices**	**Details**	**Functions**
For users			
	Bracelet	Heartbeat sensor	Data gathering
	Smartphone	Bracelet paired App	Data feedback (self-directed exercise) Data relaying
		Wechat App group	Professional feedback (supervised exercise) Peers feedback Questions and answers
For researchers			
	Computer station with internet access	Bracelet paired app	Accessing users' data
	Smartphone	Wechat App group	Sending personalized feedback (supervision) Questions and answers

For the integration of weight management, dietary recommendations and a reference menu were given at baseline to promote a low-fat low-calorie diet. However, monitoring of diet was not arranged.

### Control Group

All participants were recruited and managed in the same way as the intervention group as if we just launched our second round of weight program. But this time the online chatroom was only for the questions and answers purpose. Researchers in this chatroom usually stayed mute and did nothing of trying to intervene in their exercise. Participants in the control group did not realize they were treated as the control because they were blinded, which means they do not know what the intervention really looks like. They exclusively depended on the heartbeat tracker to do self-directed exercise as prescribed, as most wearable device buyers will do.

### Outcomes

The primary outcomes were exercising performance (exercising day, effective exercising day, and rate of effective exercising) and weight loss, and the secondary outcomes were blood pressure and lab tests.

### Data Collection

At baseline, interviews were conducted to collect demographic information. Then, physical examinations were performed, and fasting blood was collected for measuring liver enzymes, lipids, glucose, and renal function. After 12 weeks of follow-up, the same examinations were repeated. All measurements were conducted in the hospital by following standard procedures. Daily exercise data were retrieved from cloud services.

### Data Analysis

The Kolmogorov–Smirnov test was used to check the normality of the distribution of numeric variables. Data are expressed as mean (SD). Differences in group mean at baseline characteristics were demonstrated using one-way ANOVA in continuous variables and the chi-square test in categorical variables. To determine any differences between study groups after intervention analysis of covariance (ANCOVA) was used. A significant level was set at 0.05 for all comparisons. All statistical analyses were performed by using SPSS 19.0 software.

## Results

### Characteristics of Participants

**Figure 1:**
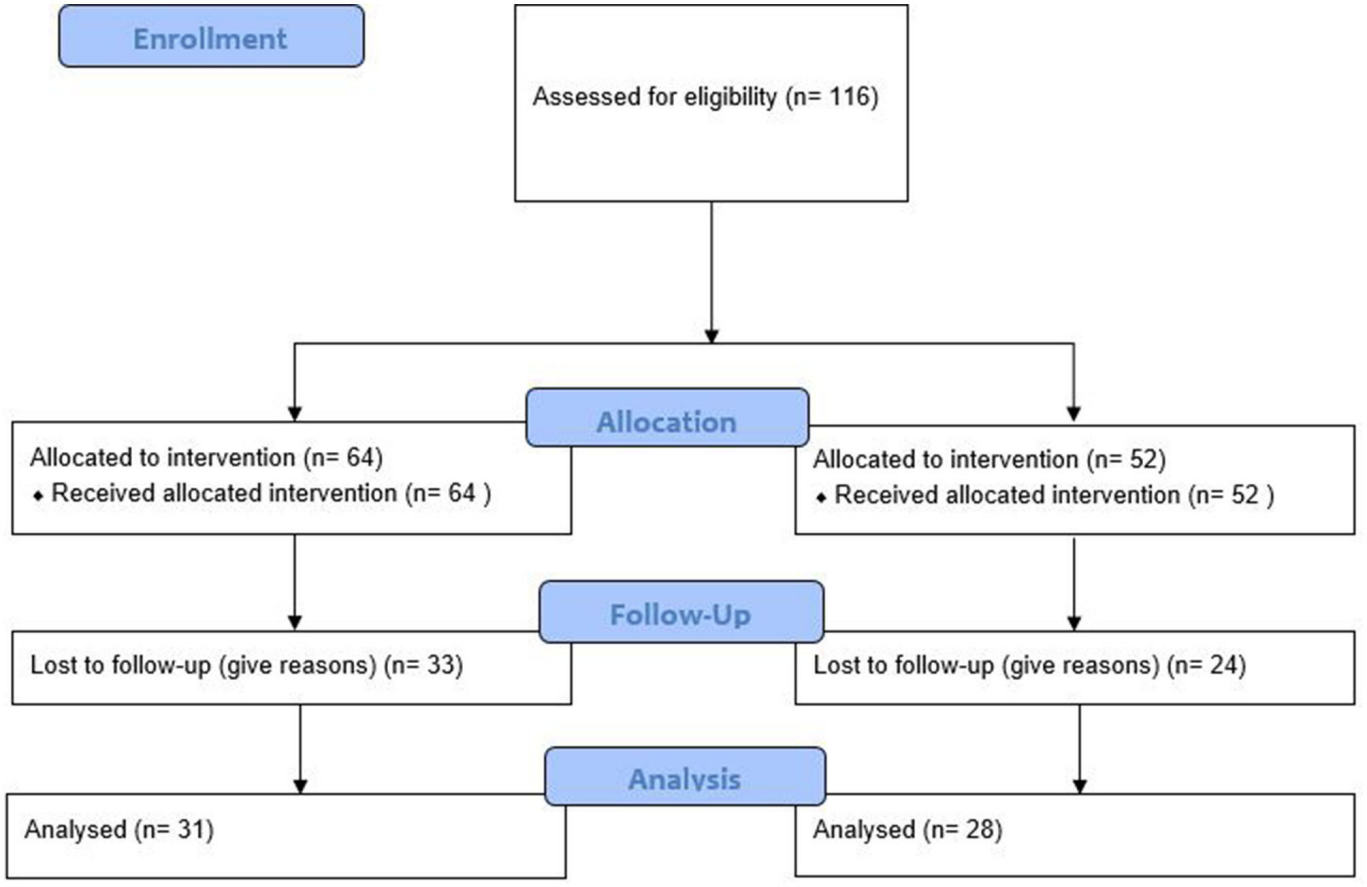
Study flow diagram.

All participants were residents of Chongqing city. They usually were well educated and savvy about kinds of smart devices due to their popularity in all metropolitan cities in China, Chongqing included. The trial design and flowchart are presented in Figure 1. The proportion of participants who completed trials in the intervention group (51.6%) and the control group (46.2%) was not different (*P* = 0.562). Younger and higher BMI participants in the intervention group were more likely to lose follow-up (both *P* < 0.05). The sex, age, height, weight, and BMI of participants who survived trials in two groups were not different (all *P* > 0.05) (Table 2).

**Table 2:** Characteristics of participants.

	**Intervention group**		**Control group**		***P1***	***P2***	***P3***
	**Lost (*n* = 33)**	**Stay (*n* = 31)**	**Lost (*n* = 24)**	**Stay (*n* = 28)**			
sex					0.119	0.103	0.779
Male (*n*,%)	17 (63.0%)	10 (37.0%)	14 (58.3%)	10 (41.7%)			
Female (*n*,%)	16 (43.2%)	21 (56.8%)	10 (35.7%)	18 (64.3%)			
Age (year)	32.3 ± 9.1	38.3 ± 8.5*	39.3 ± 9.6	40.8 ± 8.7	0.012	0.539	0.266
Height (cm)	165.9 ± 9.6	165.1 ± 10.2	164.9 ± 9.1	161.7 ± 7.0	0.755	0.156	0.200
Weight (kg)	81.69 ± 16.3	75.4 ± 13.0	78.6 ± 12.6	73.2 ± 8.6	0.096	0.072	0.569
BMI (kg/m^2^)	29.5 ± 4.1	27.5 ± 2.4*	28.8 ± 3.2	27.9 ± 2.0	0.021	0.234	0.389

*P1, compared stay with loss in the intervention group; P2, compared stay with loss in the control group; P3, compared stay between intervention and control, BMI = weight(kg)/square[weight(m)]*.

### Exercising Performances

**Figure 2:**
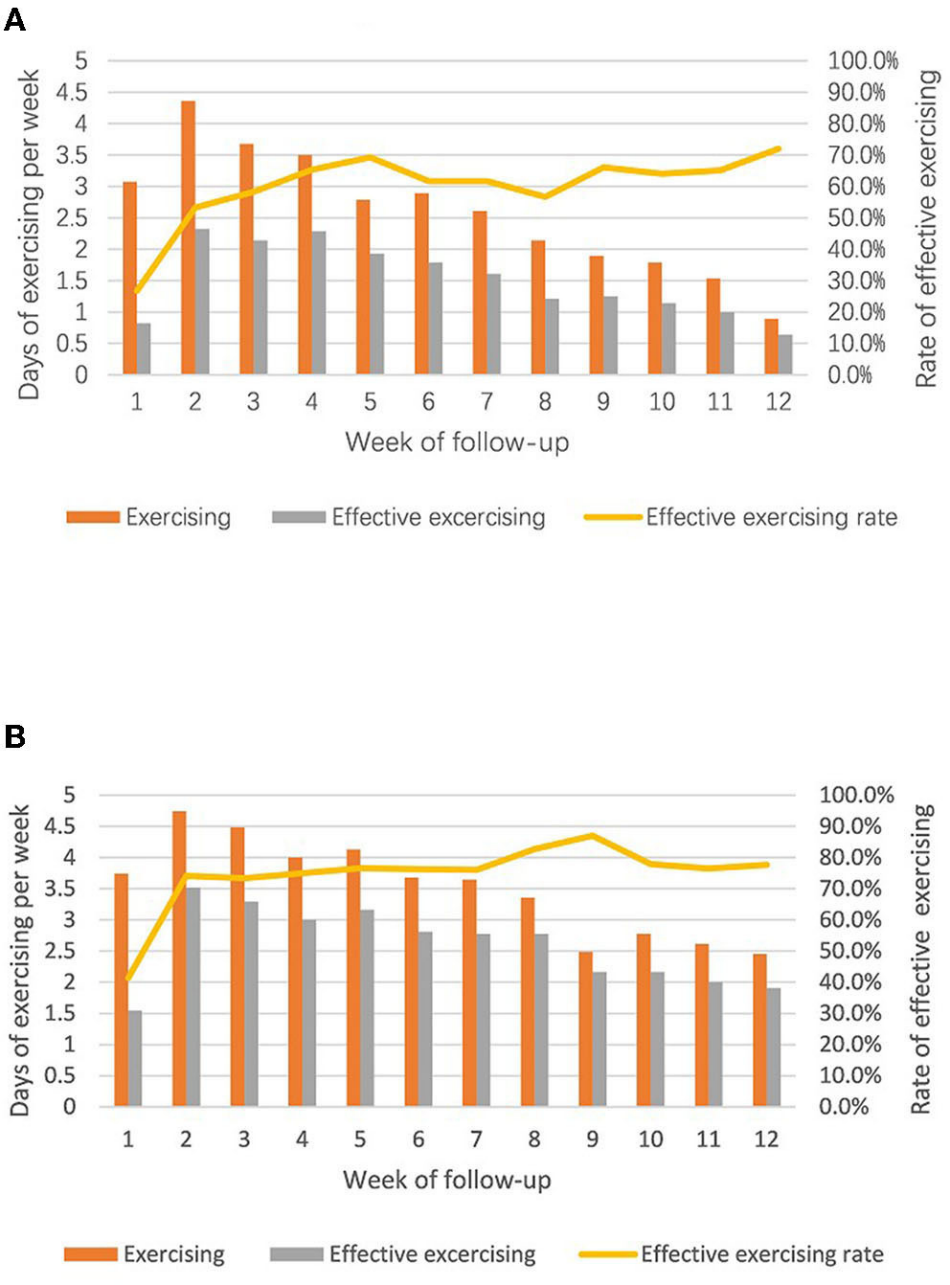
Comparisons of exercising performance between two groups **(A)** control group, unsupervised; **(B)** intervention group, supervised.

Weekly exercising day and weekly effective exercising day reached the peak in the second week and then attenuated by time in both groups with nearly the same pace, but participants in the intervention group had better exercise performances in all three aspects than the control groups (Figure 2). The average weekly exercising day, weekly effective exercising day, and the rate of effective exercising day in 12 weeks in the intervention group were 3.5 ± 0.8, 2.6 ± 0.6, and 74.6 ± 11.1%, respectively, which were higher than that (2.6 ± 1.0, 1.5 ± 0.6, and 60.0 ± 11.7%, respectively) in the control group (all *P* < 0.05).

### Weight Losses

Weight loss was significantly in each group compared with baseline (all *P* < 0.01) (Table 3), but the average weight loss was not significant between the intervention group (2.7 ± 2.8 kg) and the control group (2.0 ± 2.9) (*P* = 0.23). The average change in BMI was not significant between two of the groups too (−0.9 ± 1.0 kg/m^2^ in the intervention group and −0.7 ± 1.0 kg/m^2^ in the control group; *P*-value: 0.29). When classified weight loss over 5% as having clinical significance (17), there were 41.9% of participants in the intervention group lost weight with clinical significance, compared to 21.4% in the control group (*P* = 0.07).

**Table 3:** Weight changes after 12 weeks of follow-up.

	**Intervention**	**Control**	***P#***
	***n* = 31**	***n* = 28**	
Weight change (kg)	−3.3 ± 2.5*	−1.5 ± 2.8*	0.009
BMI (kg/m^2^)	−1.3 ± 0.9*	−0.5 ± 1.0*	0.004
Over 5% weight loss (*n*,%)	13 (41.9%)	6 (21.4%)	0.079

*Compared with 0 at baseline (p <0.001); #compared between intervention and control. BMI = weight(kg)/square[weight(m)].

### Other Health Outcomes

**Figure 3:**
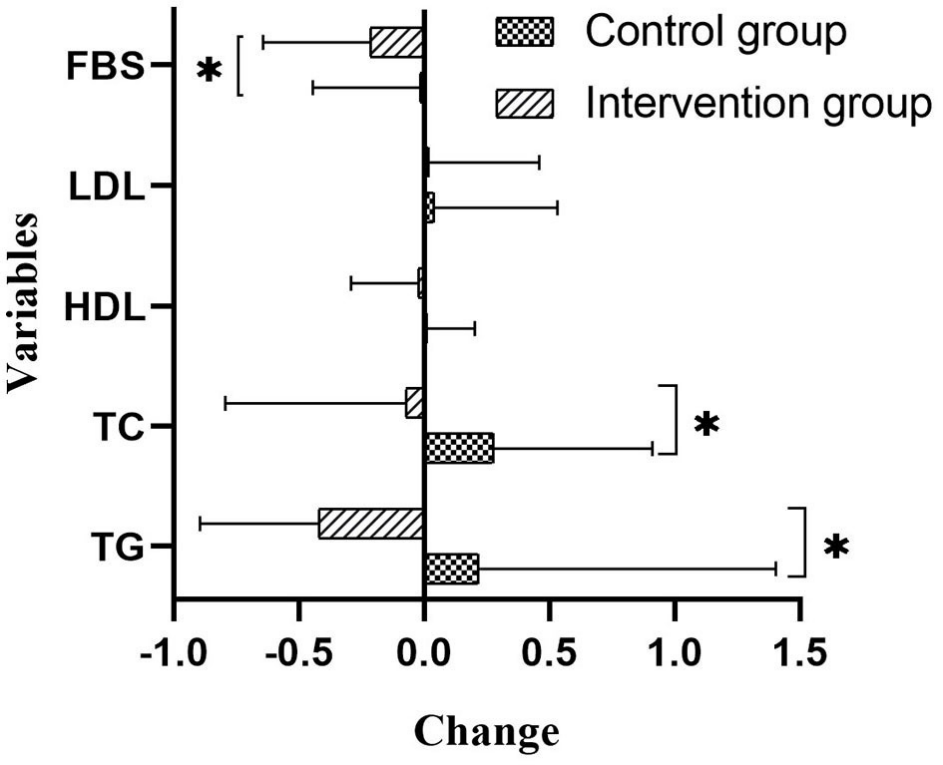
Differences of Fasting blood lipids and glucose between intervention and control groups (mmol/L). Compared between intervention and control groups with ANCOVA test and adjusting for the age, sex, and baseline amounts. FBS, fasting blood sugar; LDL, low density lipoprotein; HDL, high density lipoprotein; TG, triglyceride.

Compared with the control group, participants in the intervention group improved their liver function (*P* < 0.05 for four liver enzymes) and kidney function (*P* < 0.05 for total bile, uric acid, and creatinine) (Table 4). Figure 3 provided differences in fasting blood lipids and glucose change between intervention and control groups. Fasting blood sugar (*p* value: 0.01), total cholesterol (*p* value: 0.03), and triglyceride (*p* value: 0.01) decreased in the intervention group compared to the control group, significantly (*p* < 0.05). However, there are not any significant differences in low-density lipoprotein (*p* value: 0.61) and high-density lipoprotein (*p*-value: 0.56) changes between intervention and control groups.

**Table 4:** Differences in health examinations between intervention and control groups.

	**Intervention**	**Control**	P*
	***n* = 31**	***n* = 28**	
	**Mean±SD**	**Mean±SD**	
**Blood pressure**			
SBP(mmHg)	−3.5 ± 10.5	−0.7 ± 11.1	0.335
DBP(mmHg)	−2.5 ± 8.0	−1.6 ± 7.6	0.677
**Liver function**			
ALP, u/L	−3.8 ± 6.9	6.3 ± 11.9	*0.001*
ALT, u/L	−13.9 ± 31.0	1.6 ± 13.9	*0.018*
AST, u/L	−6.7 ± 15.1	2.8 ± 5.2	*0.003*
GGT, u/L	−9.2 ± 13.4	1.3 ± 9.9	*0.001*
**Kidney function**			
Total bile, μmol/L	−1.0 ± 3.1	1.5 ± 3.4	*0.005*
Total protein, g/L	−0.4 ± 4.5	−0.3 ± 3.0	0.919
Globulin, g/L	−1.0 ± 3.8	−0.2 ± 2.2	0.351
Uric acid, μmol/L	−29.9 ± 57.0	6.9 ± 39.8	*0.007*
Urea, μmol/L	0.1 ± 1.0	−0.1 ± 0.8	0.332
Creatinine, mmol/L	−6.5 ± 10.4	8.7 ± 11.8	*0.001*
**Fasting blood lipids and glucose**			
TG, mmol/L	−0.4 ± 0.5	0.2 ± 1.2	*0.009*
TC, mmol/L	−0.1 ± 0.7	0.3 ± 0.6	0.058
HDL, mmol/L	0.0 ± 0.3	0.0 ± 0.2	0.563
LDL, mmol/L	0.0 ± 0.4	0.0 ± 0.5	0.863
FBS, mmol/L	−0.2 ± 0.4	0.0 ± 0.4	0.082

*Compared between intervention and control groups.

## Discussions

The supervised exercise which was usually conducted on-site has been proven to be effective compared with nonsupervised exercise on weight loss, glycemic control, claudication, or improved body composition (6–8, 18). But this type of face-to-face supervision is costly and resource-intensive (12), which limits its application to overweight and obese individuals, who usually live in communities. On the contrary, remote supervision can be realized through wearable devices and modern communication technologies with more flexibility and resource-saving. Therefore, the effects of remote supervision on the exercise of overweight and obesity need to be evaluated before scaling up for weight control. Despite many studies provided online professional support based on behavior change theories, such as goal setting, education, reminder, and instruction, etc., and showed positive effects on weight loss (19), those online supports were given either without monitoring (20) or based only on a diary to report performances (21) which lacks in time-sensitivity. Some mHealth studies which showed short-term efficacy in weight loss were conducted with minimal or standard care (11, 12). In fact, studies which had directly compared the mHealth-based remote supervision to mHealth-based self-directed exercise are scarce (10). Therefore, the effects of remotely supervised exercise were far away of being well documented.

We at the baseline gave both groups a personalized exercise prescription for each of the participants and required them to wear heartbeat trackers which can feedback them to follow exercise prescriptions. In addition, researchers supervised exercise in the intervention group by checking performances daily, giving participants instructions, reminders, and encouragements accordingly. Our study showed that nearly 50% of participants lost in 12 weeks of follow-up in both groups, which was similar to the study of Lindner et al. (22). This study further confirmed that adhering to exercise is a big challenge in both groups, and even supervision had limited impacts on adherence. Participants who dropped out in our study may be not ready to do enough exercise for weight loss according to the stages of change theory (SCT) which was proposed by Prochaska (23). For those who completed the trial, the curve of exercise performance by the time was similar to the peak at the second week in both groups, which may indicate the effects of supervision are highly dependent on self-motivated exercise as was shown in the control group, i.e., if someone was self-motivated to do exercise, remote supervision will make them do it in a better way.

Usually, physical activity trackers were sold for people to do self-directed and nonsupervised exercise with or without peer competition or peer support. But Mitesh S. Patel believed that this type of exercise engagement may be easy for the already motivated and quantified-self audience but is likely to be difficult for a large group of people with difficult-to-manage chronic health conditions (13). Supervised exercise, no matter whether on-site or remote, will give people an awareness of being under observation which has a positive influence on behavior (24). Furthermore, supervision can also provide personalized feedback and skills which were believed critical for behavior-changing (25, 26). Therefore, supervised mHealth exercise may exert more profound effects on sustaining prescribed exercise than nonsupervised mHealth exercise.

Even so, our findings suggested that remote supervision could not motivate more people to complete trials but was only useful to those who had already been motivated to engage in regular exercise for at least 3 months. As maintaining long-term exercise is critical for weight control, whether the remotely supervised exercise can keep more people who survive 3 months trial engaging in exercise over 3 months than the wearable device-based self-directed exercise is warranted.

To our best knowledge, this study is the first time to compare the effects of mHealth-based remotely supervised exercise with mHealth-based self-directed exercise among overweight and obesity. This study provided solid evidence with proper control and enough sample size to assess the effects of mHealth-based remotely supervised exercise. But still, there were some limitations in this study. First, this study is a nonrandomized trial which may introduce bias in selection, but the baseline characteristics were comparable between the two groups which indicated selection bias is not obvious. Second, two arms of the trial were tested in tandem, therefore influences of season and implementation seem unavoidable. Third, the intervention duration was only 12 weeks, and the long-term effects of remote supervision are still uncertain. Last, as different supervision regimes and population characteristics may lead to different responses to exercise intervention (11), our results based on middle-aged volunteers with heartbeat tracking may not be generalized to other supervision regimes, wearable devices, and populations.

Physical activity tracker-based remotely supervised exercise can be introduced into a health and exercise program to enhance the effects of wearable devices-based self-directed exercise for overweight and obesity. But further studies are warranted to test the ability of wearable tracker-based supervision to maintain long-term exercise engagement.

## Data Availability Statement

The raw data supporting the conclusions of this article will be made available by the authors, without undue reservation.

## Ethics Statement

The studies involving human participants were reviewed and this study is a prospective, non-randomized controlled study approved by Ethics Committee of Chongqing Medical University (s2018-065-01). The patients/participants provided their written informed consent to participate in this study.

## Author Contributions

All authors listed have made a substantial, direct, and intellectual contribution to the work and approved it for publication.

## Funding

This research was funded by the following: Chongqing Medical Scientific Research Project (Joint project of Chongqing Health Commission and Science and Technology Bureau) (NO: 2021MSXM057); Humanities and Social Sciences Research Project of Chongqing Municipal Education Commission (NO: 22SKGH065). The funders had no role in study design, data collection and analysis, decision to publish, or preparation of the manuscript.

## Conflict of Interest

The authors declare that the research was conducted in the absence of any commercial or financial relationships that could be construed as a potential conflict of interest.

## Publisher's Note

All claims expressed in this article are solely those of the authors and do not necessarily represent those of their affiliated organizations, or those of the publisher, the editors and the reviewers. Any product that may be evaluated in this article, or claim that may be made by its manufacturer, is not guaranteed or endorsed by the publisher.
